# Preclinical 3D-model supports an invisibility cloak for adenoid cystic carcinoma

**DOI:** 10.1038/s41598-023-44329-7

**Published:** 2023-10-09

**Authors:** Rajdeep Chakraborty, Charbel Darido, Arthur Chien, Aidan Tay, Karen Vickery, Honghua Hu, Fei Liu, Shoba Ranganathan

**Affiliations:** 1 Applied Biosciences, Faculty of Science and Engineering, Macquarie University, Sydney, NSW 2109 Australia; 2 Peter MacCallum Cancer Centre, Melbourne, VIC 3000 Australia; 3 Sir Peter MacCallum Department of Oncology, The University of Melbourne, Melbourne, VIC 3000 Australia; 4 School of Natural Sciences, Faculty of Science and Engineering, Macquarie University, Sydney, NSW 2109 Australia; 5 Macquarie Medical School, Faculty of Medicine Health and Human Sciences, Macquarie University, Sydney, NSW 2109 Australia

**Keywords:** Cancer, Immunology, Oncology, Pathogenesis

## Abstract

The tumour-cell based initiation of immune evasion project evaluated the role of Gipie in adenoid cystic carcinoma (ACC) and mucoepidermoid carcinoma (A-253), from ninety-six 3D-ACC and A-253-immune co-culture models using natural killer cells (NK), and Jurkat cells (JK). Abnormal ACC morphology was observed in 3D-ACC immune co-culture models. Gipie-silencing conferred a “lymphoblast-like” morphology to ACC cells, a six-fold increase in apoptotic cells (compared to unaltered ACC cells, *P* ≤ 0.0001), a two-fold decrease in T regulatory cells (FoxP3^+^/IL-2Rα^+^/CD25^+^) (*P* ≤ 0.0001), and a three-fold increase in activated NK cells (NKp30^+^/IFN-γ^+^) (*P* ≤ 0.0001) with significantly higher release of granzyme (*P* ≤ 0.001) and perforin (*P* ≤ 0.0001).

## Introduction

Gipie (*CCDC88B*) is a member of the Hook-related protein family (cytoplasmic organelle to microtubule linker proteins)^1^. Gipie participates in T cell maturation and inflammatory processes^1^. Gipie has also been involved in mobility and inflammatory functions of dendritic cells^2^. Gipie is also involved in the regulation of microtubule-mediated lytic granule clustering and subsequent target cell killing in natural killer (NK) cells^3^. In addition to this, Genome-Wide Association Studies discovered that mutations in the Gipie coding gene (*CCDC88B*) are strongly associated with susceptibility to inflammatory and immune diseases such as sarcoidosis, inflammatory bowel disease (IBD) and leprosy^4^. Surprisingly, we found its expression in adenoid cystic carcinoma (ACC) cells, albeit without known functionality (Supplementary Fig. 1). We hypothesized that Gipie affects the survival of ACC cells.

We constructed a 3D-ACC-immune cell co-culture model using UM-HACC-2A (adenoid cystic carcinoma cells), (which are generated from human ACC tumour that demonstrated a circumscribed collection of myoepithelial cells with a distinctive cribriform pattern characteristics of ACC) that exhibits epithelial morphology and high expressions of epithelial markers (E-cadherin, EGFR, pan-cytokeratin) and myoepithelial markers (P63)^5^, along with another salivary gland cancer 3D-preclinical cellular model, from mucoepidermoid carcinoma (A-253) cells. The apoptotic effect was assessed by annexin V flow cytometry. Gipie’s role in immune modulation was investigated by assessing T regulatory (Treg) cells, activated NK cells, and immune cell transmigration towards cancer cells (Supplementary Fig. 2).

## Methods

Optimized salivary gland medium was used to grow UM-HACC-2A (Cat T8326) (abm). A-253 (Cat HTB-41) (ATCC) and additional oral cancer cells were used to construct multiple 3D head and neck cancer models. Silencing of the cancer cells was performed for 48 h. Jurkat, Clone E6-1(Cat TIB-152) (ATCC) and NK-92 (CRL-2407) (ATCC) cells were the immune cells that were used to construct the 3D models. The immune cells-cancer cells interaction was maintained for 16 h. Cell culture inserts acted as an interface between cancer and immune cells (Supplementary Table 1). Cell culture insert 24 well, 0.4 µm (Cat NUN140640) (Thermo Fisher Scientific), Cell culture insert 6 well, 3 µm PET membrane (Cat FAL353091) (Falcon), and Cell culture insert 6 well, 0.4 µm PET membrane (Cat FAL353090) (Falcon) was used to construct 3D cancer-immune co-culture model. Cancer cell adherence was facilitated by coating the compartments with Fibronectin human plasma (F0895-2MG) (Sigma Aldrich). Interferin (siRNA transfection reagent) (Cat 101000028) (Polyplus) and 1 nmol *CCDC88B* silencer pre-designed siRNA (Cat AM16704) (Ambion) was used for silencing Gipie.

To understand the effect of Gipie on ACC and its corresponding immune cells; apoptosis, Treg, and NK cell activation assays were performed via flow cytometry. TACS Annexin V-FITC Apoptosis detection kit (RDS482001K) (R&D Systems) was used for apoptosis detection. T regulatory cells were detected by FlowX human regulatory T cell multi-colour flow kits containing IL-2Rα/CD25-APC conjugated, CD4-FITC conjugated, FoxP3-PE conjugated antibodies (RDSFMC021) (R&D Systems). NK activation was detected by NKp30/NCR3-PE conjugated antibody (FAB1849P-025) (R&D Systems) and IFN-γ-Alexa Fluor 488 conjugated antibody (IC285G-025) (R&D Systems). Flow cytometry analysis was done in FlowJo 10.8.1 (Becton Dickinson & Company, U.S.A).

Morphology, live cell imaging, and transmigration assays were captured by Olympus FV3000RS (Olympus-lifescience). Morphology and transmigration assay done after staining the cells and membranes with ActinGreen 488 (Cat R37110) (Thermo Fisher Scientific), ProLong Glass Antifade Mountant with NucBlue (Cat P36983) (Thermo Fisher Scientific), and the images were captured by using Olympus FV3000RS (Olympus-lifescience). Each morphology and live cell imaging were captured at a magnification of 100X, and using channels Alexa Fluor 488 (Excitation 488, Emission 520) and DAPI (Excitation 359, Emission 461). Generation of the 3D re-constructed images was done after acquiring 3D Z-stack image [series] that went through the 3D construction module in the Olympus software. The 3D transmigration image video recording was done in Olympus FV30S-SW at a frame rate of 15. Live image intensity profile was generated using the “Live/ROI ID/Channel” tool. The average intensity profile is drawn in synchronization with acquisition using FV31S-SW Version: 2.6.1.243 (Powered by H-PF Version 2.14.2.717).

Mass spectrometry (MS)-based proteomics data of the immune cell co-cultures were acquired following the standard operation protocol of Australian Proteome Analysis Facility (APAF) (Macquarie University, Sydney). Mass spectrometry (MS) analysis was done via ion-based protein quantification, implementing maximal peptide ratio extraction algorithm for data independent acquisition. We used triple-TOF for data independent acquisition. Heat map generation was done by RStudio 2021.09.0 Build 351.

All Statistical analysis were performed using GraphPad Prism version 9.4.1 (GraphPad Software, San Diego, CA, USA). Unpaired t test was performed to analyse flow cytometry, imaging, and MS-data. The probability threshold of P < 0.05 was considered statistically significant. All experiments were repeated at different time points. Throughout this report, *n* refers to the number of 3D salivary gland models of each experimental group. All experiments had positive, negative, isotype, and internal controls.

### Ethics statement

The entire work was done following the biosafety approval by the Macquarie University Biosafety Committee (“Mammalian Cell Culture” 5215).

## Results

**Figure 1:**
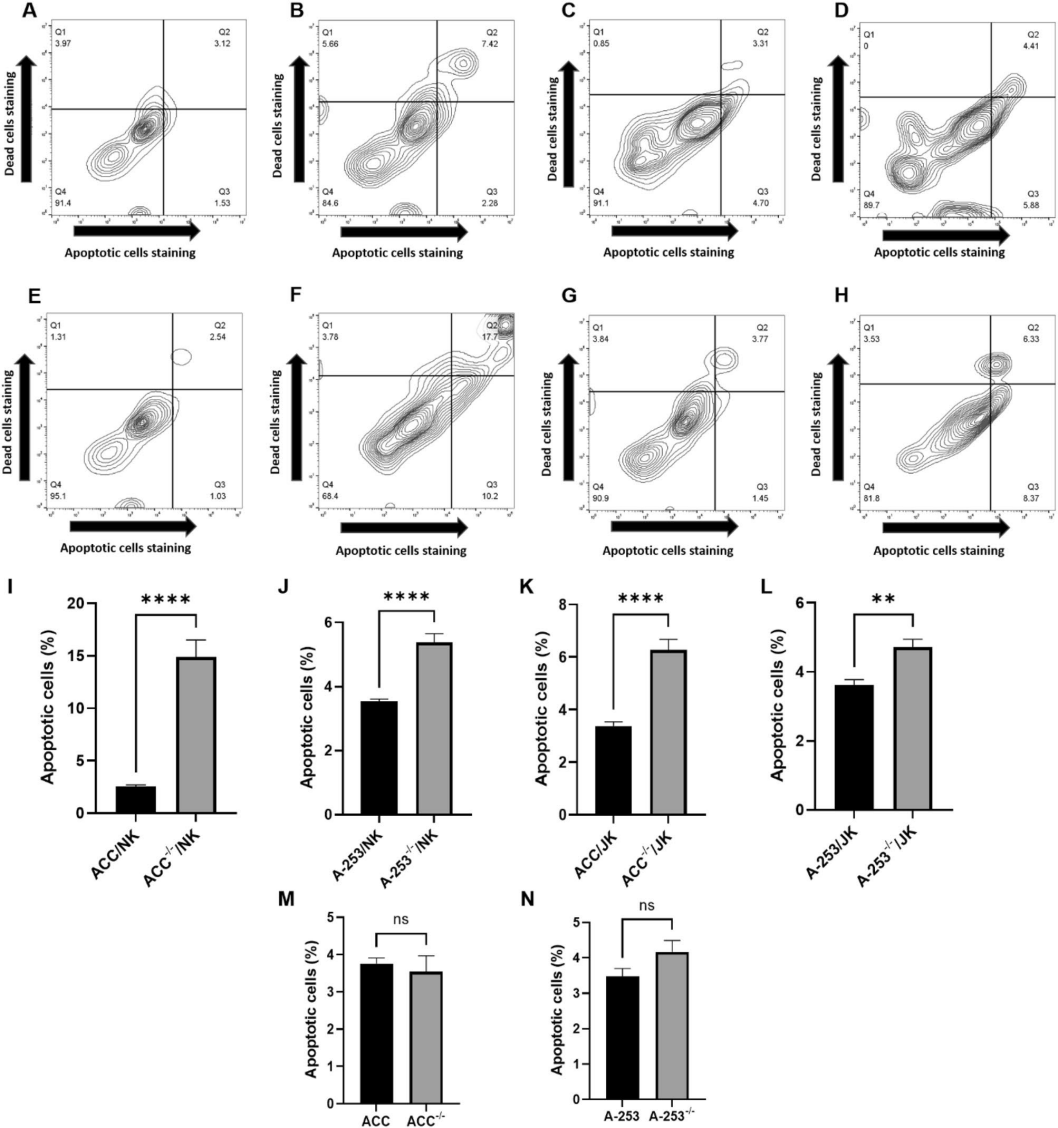
Contour plot representations of annexin V flow cytometry (**A**) ACC/JK, (**B**) ACC^−/−^/JK, (**C**) A-253/JK, (**D**) A-253^−/−^/JK, (**E**) ACC/NK, (**F**) ACC^−/−^/NK, (**G**) A-253/NK, (**H**) A-253^−/−^/NK; Graphical representations pairwise analysis of apoptotic cell percentage of 3D-salivary gland cancer models (**I**–N) (n = 24 3D-models); Graphical representations pairwise analysis of apoptotic cell percentage of salivary gland cancer cell culture models (without immune co-culture) (n = 6 cell culture models); **P ≤ 0.01, ****P ≤ 0.0001. The error bar represents the standard error of the mean.

The preliminary check was whether Gipie suppressed apoptosis in 3D-salivary gland cancer-immune co-culture models. Annexin V apoptotic flow cytometry assay revealed no significant change in the percentage of apoptotic cells in ACC cells (without immune cell co-culture), when ACC cells were compared to their Gipie-silenced subset. However, there was a nearly six-fold and two-fold increase in apoptotic ACC cells (from the 3D-ACC-immune co-culture model) in Gipie-silenced ACC from the ACC^−/−^/NK and ACC^−/−^/JK models, respectively, compared to their unaltered counterparts. A-253^−/−^/NK and A-253^−/−^/JK models showed similar but much lower results (Fig. 1).

The observed apoptotic cell content of Gipie-silenced ACC: 14.90% and A-253: 5.38% compared to 2.52% and 3.5% respectively in unaltered ACC and A-253 cells: ACC^−/−^/NK versus ACC/NK (*P* ≤ 0.0001, 95% CI 8.776, 15.99) and A-253^−/−^/NK versus A-253/NK (*P* ≤ 0.0001, 95% CI 1.221, 2.486) respectively. The results were similar albeit less dramatic with JK cells: 6.28%, 4.72%, and 3.61%, 3.38% in Gipie-silenced ACC and A-253 and unaltered ACC and A-253 cells: ACC^−/−^/JK versus ACC/JK (*P* ≤ 0.0001, 95% CI 1.949, 3.858) and A-253^−/−^/JK versus A-253/JK (*P* = 0.0024, 95% CI 0.4922, 1.718) respectively (Fig. 1).

**Figure 2:**
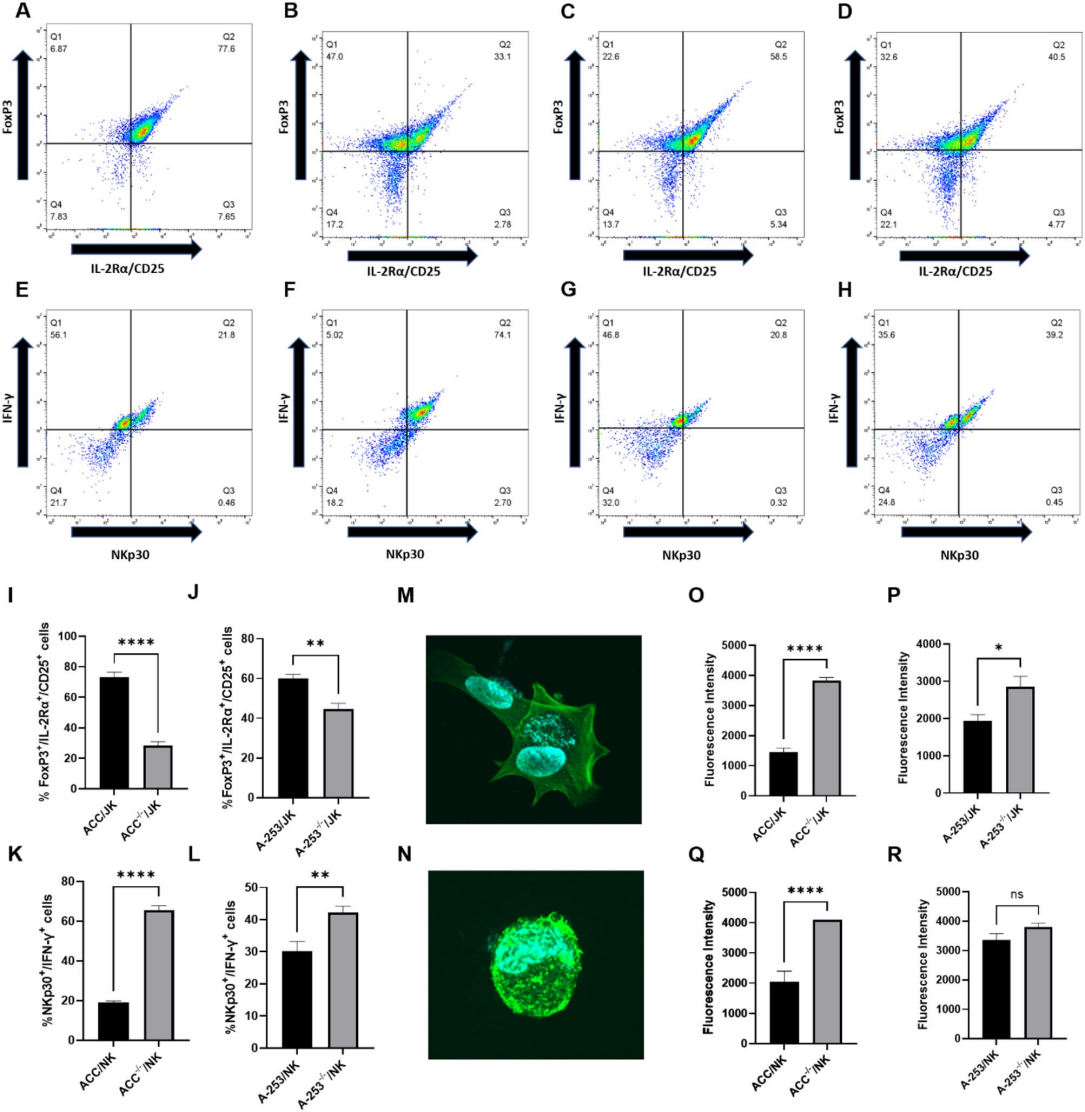
Preclinical salivary gland cancer model response to silencing Gipie. Density plot representations of T regulatory cell detection from (**A**) ACC/JK, (**B**) ACC^−/−^/JK, (**C**) A-253/JK, (**D**) A-253^−/−^/JK; density plot representations of NK activation (**E**) ACC/NK, (**F**) ACC^−/−^/NK, (**G**) A-253/NK, (**H**) A-253^−/−^/NK; graphical representations of pair-wise analysis of FoxP3^+^/IL-2Rα^+^/CD25^+^ aka Treg cells percentage (**I**,**J**); graphical representations of pairwise analysis of NKp30^+^/IFN-γ^+^ aka activated NK cells percentage (**K**,**L**); graphical representations pairwise analysis of fluorescence intensity (O-R); 3D z-stack confocal images of (M) ACC cells from 3D-ACC/NK model and (N) ACC cells from Gipie-silenced 3D-ACC^−/−^/NK model (*n* = 24 3D-models); *P ≤ 0.05, **P ≤ 0.01, ****P ≤ 0.0001. The error bar represents the standard error of the mean.

These results were corroborated by a significant decrease in T regulatory (Treg, FoxP3^+^/IL-2Rα^+^/CD25^+^) cells, which occurred after silencing Gipie. Substantial lowering of Treg cells were observed in the Gipie-silenced ACC model. 73.13% JK cells from ACC/JK were Treg compared to 28.40% Treg in the Gipie-silenced subset: ACC^−/−^/JK versus ACC/JK (*P* ≤ 0.0001, 95% CI  -54.10,  -35.36). This effect was less pronounced with A-253/JK with 59.95% JK cells from A-253/JK being Tregs compared to 44.48% JK cells in the Gipie-silenced A-253^−/−^/JK preclinical model: A-253^−/−^/JK versus A-253/JK (*P* = 0.0018, 95% CI  -23.65,  -7.288) (Fig. 2A–D,I,J).

Similar to the Treg results, higher activation of NK cells was observed in Gipie-silenced ACC^−/−^/NK models. 65.55%, 42.23% and 18.93%, 30.10% activated NK (NKp30^+^/IFN-γ^+^) cells seen in Gipie-silenced ACC^−/−^/NK, A-253^−/−^/NK and their unaltered counterparts: ACC^−/−^/NK versus ACC/NK (*P* ≤ 0.0001, 95% CI 41.11, 52.13) and A-253^−/−^/NK versus A-253/NK (*P* = 0.0079, 95% CI 3.970, 20.30) respectively (Fig. 2E,H,K,L).

Striking differences in ACC morphology were observed, when ACC cells (without immune coculture) was compared to the 3D-ACC-immune co-culture model. Many of the ACC cells from the 3D-ACC-immune co-culture model were neither spindle shaped nor fully elongated. After silencing Gipie, most of the ACC cells showed oval or round morphology, which could be classified as “lymphoblast-like” (Fig. 2M,N).

Further staining of the semipermeable membrane, harboring immune cells for actin intensity, confirmed NK cell activation, and the data further validated the immune activation results, with Gipie-silencing leading to a twofold increase in the fluorescence intensity of NK cells from the ACC^−/−^/NK model: ACC^−/−^/NK versus ACC/NK (*P* ≤ 0.0001, 95% CI 131, 2801), with no significant difference in fluorescence intensity found in A-253/NK models, following Gipie silencing. Similar JK fluorescence intensity results were seen with ACC^−/−^/JK versus ACC/JK (*P* ≤ 0.0001, 95% CI 2001, 2740) and A-253^−/−^/JK versus A-253/JK models (*P* = 0.0141, 95% CI 204.7, 1617), (Fig. 2O–R).

**Figure 3:**
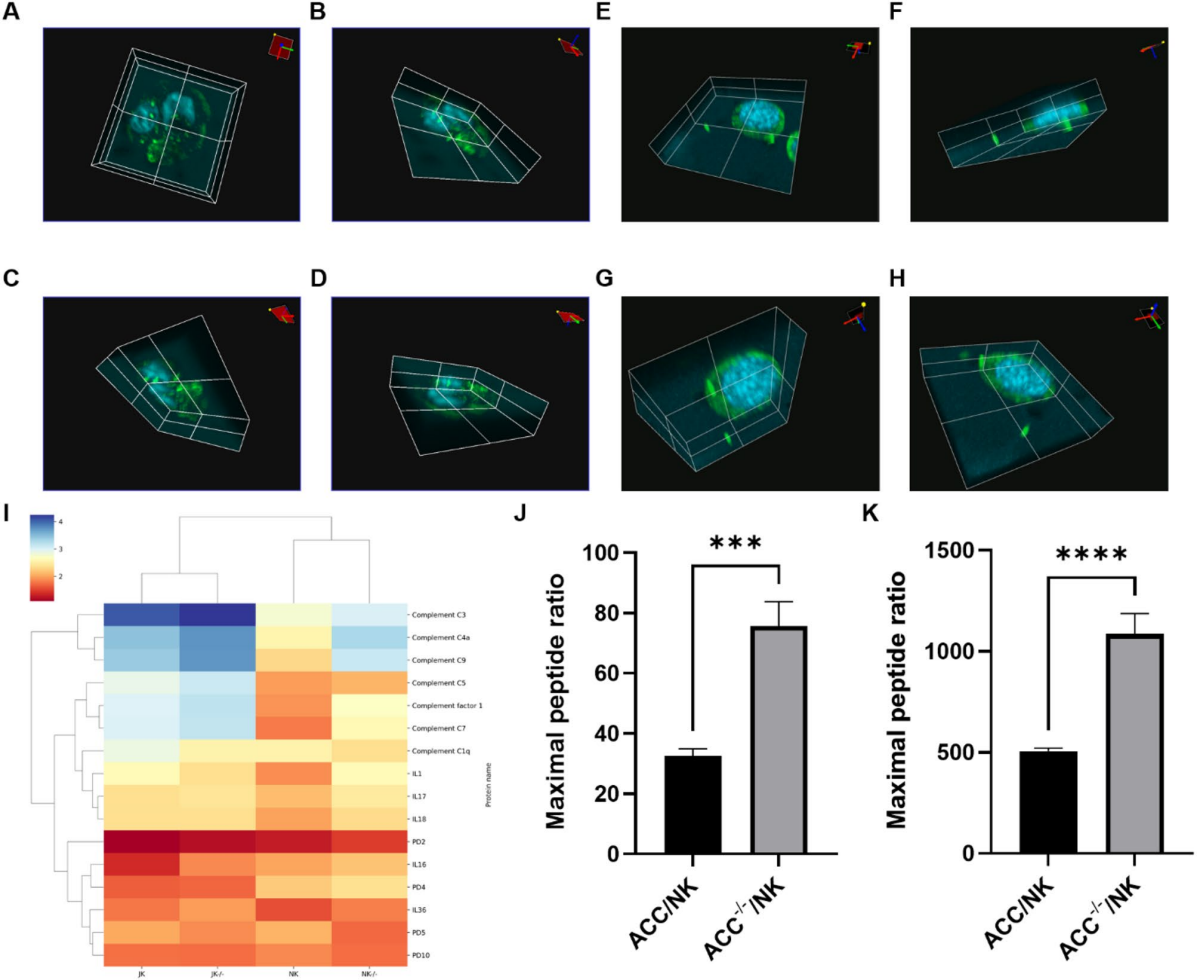
Imaging and MS analysis of the 3D ACC immune co-culture model. (**A**–**D**) Transmigration image of NK cells; (**E**–**H**) transmigration image of JK cells (*n* = 24 3D-models); (**I**) heat map representation of MS analysis of JK and NK cells from ACC 3-D model; graphical representations of pairwise analysis of (**J**) granzyme and (**K**) perforin (*n* = 12 3D-models); ***P ≤ 0.001, ****P ≤ 0.0001.

3D-z-stack confocal images of the NK and JK cells (harboured in the semipermeable membrane) from the 3D ACC models clearly exhibited transmigration of immune cells to the ACC compartment, with immune cells showing increased incidences of transmigration in Gipie-silenced models (Fig. 3A–H) (Supplementary videos 1 and 2).

MS-analysis was conducted to validate the above immune cell activation. We report, that the maximal peptide ratios of complement components (3, 1, 4a, 5, 7, 9), interleukins (IL1, IL16, IL17, IL18, IL36), and programmed cell death proteins (PD2, PD4) were higher in both NK and JK from Gipie-silenced cancer-immune co-culture subsets, with the converse seen in complement components C1q, PD5 (PDCD5), and PD10 (PDCD10) (Fig. 3I). Similarly, significant increase in the maximal peptide ratio of granzyme, 214%: ACC^−/−^/NK versus ACC/NK (*P* ≤ 0.001, 95% CI 359.9, 798.1) and perforin, 232%: ACC^−/−^/NK versus ACC/NK (*P* ≤ 0.0001, 95% CI 25.28, 60.92) (Fig. 3J,K).

## Discussion

Following apoptosis and immune activation flow cytometry assays, we found that Gipie-silencing of ACC cells, increases apoptotic ACC cells in the presence of immune cells. Treg cells were significantly lower, but activated NK cells were significantly higher in Gipie-silenced salivary gland cancer models. An increase in NKp30^+^/ IFN-γ^+^ NK cells is implicated in the cytotoxicity of NK cells^6^. Additionally, high intensity of actin^7^, higher expression of perforin^8^, and granzyme^9^, (signifying NK cell activation) and higher incidences of transmigration, cumulatively suggest higher activation of immune cells in Gipie-silenced salivary gland cells. Thus, a preponderance of evidence indicates that there is a causal effect between Gipie and immune attack.

Platelet-derived MHC class I conferred a pseudonormal phenotype to cancer cells that avoided killing by natural killer (NK) cells^10^. Similarly, here we found an undefined morphology of ACC in the 3D-ACC-immune co-culture model. Ostensibly the common morphologies of a dividing or differentiating cells are either spindle-shaped or elongated. Paradoxically, ACC morphology, which was captured at a confluency of lower than 80%, was difficult for us to describe. In contrast, after silencing Gipie, in the ACC, the morphology was transformed to a round or oval shape. The change of morphology due to the presence of immune proteins might be because of the changes in the actin cytoskeleton. A plethora of studies emphasise the active role of microtubule linker proteins facilitating proper organisation of the actin cytoskeleton^11^. TGFβ has previously been shown to affect the actin cytoskeletal reorganisation^12^. Both immune cells and cancer cells have previously demonstrated the release of TGFβ, that on one side dampens the immune cell activity against cancer and on other side bring changes to the actin cytoskeleton of the cancer cells, conferring a change of cancer cell morphology^13^.

Interestingly, JK cell models and the A-253 preclinical models produced fewer apoptotic cells than NK and ACC preclinical models. Again, this finding suggests a stronger role of Gipie in ACC, compared to A-253.

Additionally, Treg cell percentage in unaltered A-253 co-culture model was significantly lower than that in unaltered ACC model. Furthermore, activated NK cell percentage in unaltered A-253 co-culture model was significantly higher compared to activated NK cell percentage in unaltered ACC model. This observation was later confirmed by fluorescence intensity of the immune cells from the respective experimental groups. This indicated that immunosuppressive cells that are responsible for dampening anti-tumour reactivity are more activated in unaltered adenoid cystic carcinoma cellular model compared to mucoepidermoid carcinoma cellular model. Similarly, cytotoxic immune cells in ACC are less activated compared to MEC. The observation is concordant with previous findings supporting mucoepidermoid carcinoma being more responsive to immunotherapy compared to adenoid cystic carcinoma^14^.

Both mucoepidermoid carcinoma and adenoid cystic carcinoma have fewer treatment options. The advent of immune check point inhibitors triggered clinical trials using immunotherapy to reduce the progression of salivary gland cancers. Pembrolizumab which is a PD-1 inhibitor has shown promising clinical results in case of mucoepidermoid carcinoma^14^. Whereas pembrolizumab is still not FDA approved for the treatment of adenoid cystic carcinoma due to lack of PD-1 expression^15^. When pembrolizumab is combined with other anti-cancer drugs, it still failed to generate appreciable treatment response in ACC^16^. Indeed, a recent report suggested prolonged response to immune check point inhibitor therapy in salivary gland mucoepidermoid carcinoma^17^.

We further validated our preliminary data by proteomics analysis of immune cells from the 3D-ACC model. We found a significant increase in complement C3, which plays a central role in immune cell activation^18^ and other complement factors. An increase in IL1, IL16, IL17, IL18, and IL36 in the immune cells from the Gipie-silenced cohort further emphasizes the role of Gipie in ACC, dampening immune-mediated cell killing^19^.

Although PD5 inhibits renal cancer cell proliferation by enhancing T cell activation^18^, the decrease in Gipie-silenced PD5 could be attributed to Gipie’s role in trafficking PD5. However, PD10 depletion has been reported to induce apoptosis and decrease proliferation in malignant T cells^20^, which is concordant with the decrease in Gipie-silenced PD10 in ACC^21^, providing a potential therapeutic opportunity.

Overall, the bizarre morphological change which we have termed “invisibility cloak” of ACC becoming “pseudonormal” following Gipie-silencing and the shifting of the balance from inactive to active immune cell-mediated killing of ACC cells, are definitive. The molecular biological aspect of the action of Gipie in ACC or salivary gland cancer is still elusive. We continue conducting experiments to determine the protein–protein interactions and explore additional proteins in ACC that help immune evasion, to ultimately unlock this mystery.

### Supplementary Information

Supplementary Video 1.

Supplementary Video 2.

Supplementary Information 1.

## Data Availability

The datasets generated and/or analysed during the current study are available in the figshare repository, https://doi.org/10.6084/m9.figshare.22015487.
