# Supplementary material for: Preclinical 3D-model supports an invisibility cloak for adenoid cystic carcinoma
Source: Sci Rep. 2023 Oct 9;13:17033. doi: 10.1038/s41598-023-44329-7 (PMC10562364; doi:10.1038/s41598-023-44329-7)
Supplement: Supplementary file 3 — Supplementary Information 1. [file 41598_2023_44329_MOESM3_ESM.docx]

**Supplementary**

**Preclinical 3D-model supports an invisibility cloak for Adenoid Cystic Carcinoma**

Rajdeep Chakraborty^1*^, Charbel Darido^2,3^, Arthur Chien^4^, Aidan Tay^1^, Karen Vickery^5^, Honghua Hu^5^, Fei Liu^4^, & Shoba Ranganathan^1^

^1^Applied Biosciences, Faculty of Science and Engineering, Macquarie University, Sydney, NSW 2109, Australia

^2^Peter MacCallum Cancer Centre, Melbourne, VIC 3000, Australia

^3^Sir Peter MacCallum Department of Oncology, The University of Melbourne, Melbourne, VIC 3000, Australia

^4^School of Natural Sciences, Faculty of Science and Engineering, Macquarie University, Sydney, NSW 2109, Australia

^5^Macquarie Medical School, Faculty of Medicine Health and Human Sciences, Macquarie University, Sydney, NSW 2109, Australia

*Correspondence: [rajdeep.chakraborty@hdr.mq.edu.au](mailto:rajdeep.chakraborty@hdr.mq.edu.au)


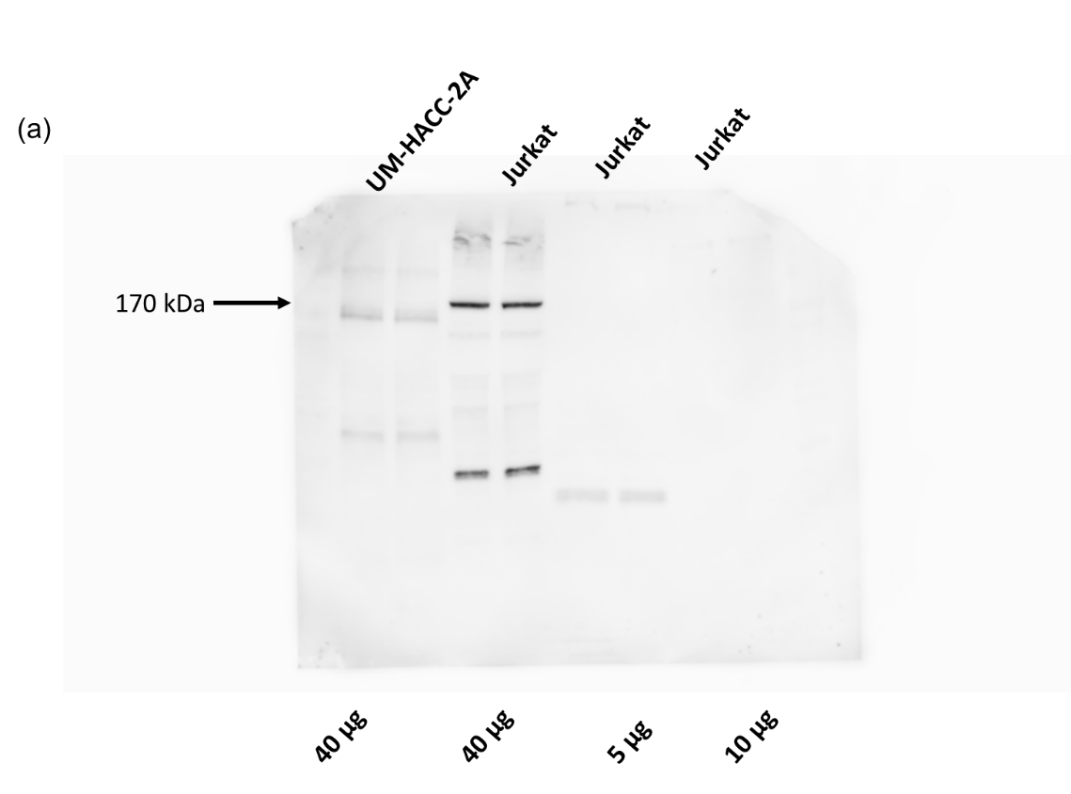


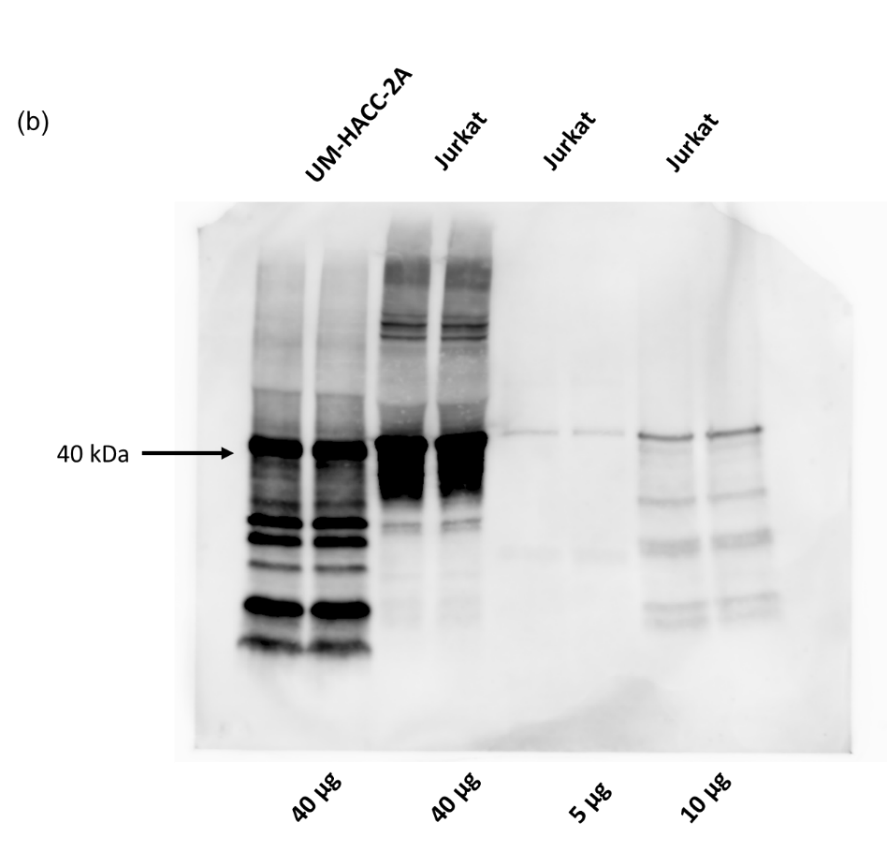


**Supplementary Figure 1. Western Blot image of UM-HACC-2A and Jurkat showing expressions of gipie.** UM-HACC-2A and Jurkat. The loading amount of UM-HACC-2A was 40µg. The loading amount of Jurkat ranged from 5 µg to 40 µg. (a) Gipie band at 170 kDa; (b) GAPDH at 40 kDa. Gipie western blot saining done with Anti-CCDC88B antibody (1:400) and GAPDH Affinity Purified Polyclonal Ab (1 µg/ml). 1536 pixels x 1024 pixels, resolution 96 dpi tiff images were acquired with Image Reader LAS-3000 version 2.1, Fujifilm.


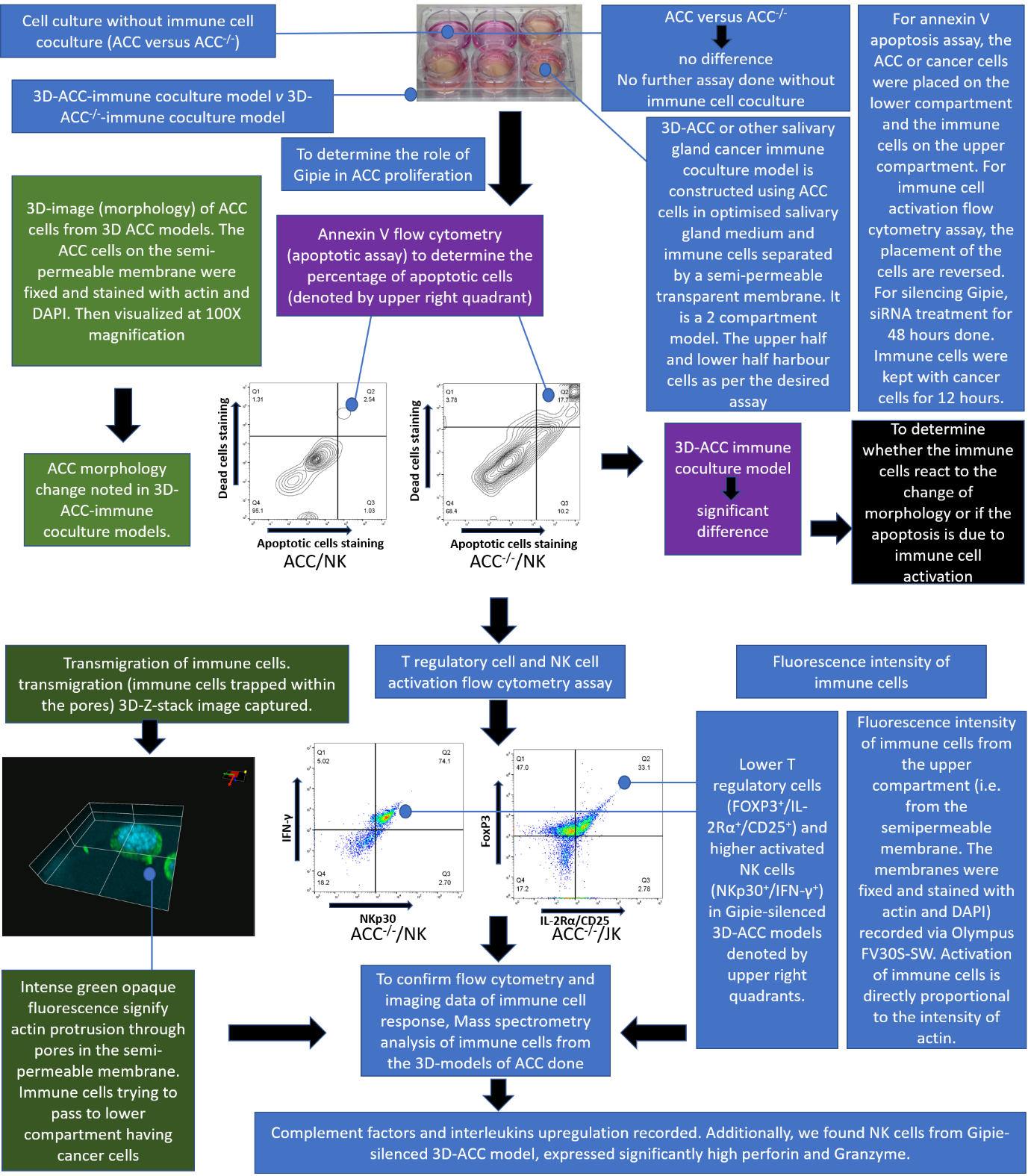


**Supplementary Figure 2.** Overview of the project. **Title:** Preclinical 3D-model supports an invisibility cloak for Adenoid Cystic Carcinoma. **Innovation:** Construction of 3D-Adenoid Cystic Carcinoma model allowed us to assess the immune cell activity during cancer cell interaction. **Outputs:** (1) ACC cells showed different morphology after interaction with immune cells in 3D model. (2) Gipie-silenced ACC cells transformed to “lymphoblast-like” morphology after interaction with immune cells in 3D model. (3) Gipie-silenced ACC cells showed significantly higher apoptosis to unaltered ACC cells in the 3D model (i.e. after immune cell interaction). No difference of apoptosis seen in cell culture (without immune coculture). (4) Immune cells from 3D model of Gipie-silenced ACC cells showed significantly lower Treg and higher activated NK cells. (5) We visualized the increased activation of immune cells via transmigration 3D stacked images. (6) Increased immune cell activity was further validated by MS-analysis showing significant increase of complement factors, perforin and granzyme. **Clinical Significance:** Presence of immune cell activating protein in ACC cells is an alarming concern. This project acts as a prelude to future study on the role of Gipie and other unknown proteins that may act as confounding factor during the cancer-immune cell interaction during conventional chemotherapy or monoclonal immunotherapy. **Limitation:** Lack of in-depth analysis of the interaction of ACC with immune cells in *in vivo* and patient tissue samples. We are still in dark regarding the role of other hook-related proteins in ACC and other salivary gland cancers. **Future Direction:** To elucidate the intersection of cancer-immune cell, we are developing *D melanogaster* ACC models.

**Supplementary Video 1.** Transmigration videos captured at 100X magnification. Here the Z stack high resolution images were combined to show the transmigration of activated Jurkat cells towards cancer cells. The green opaque protrusion is the actin cytoskeleton of the Jurkat cells towards the pores of the semi permeable membrane. The cells were fixed on the semi permeable membrane of the cell inserts by 4% paraformaldehyde and stained with DAPI (blue).

**Supplementary Video 2.** Transmigration videos captured at 100X magnification. Here the Z stack high resolution images were combined to show the transmigration of activated Natural killer cells towards cancer cells. The green opaque protrusion is the actin cytoskeleton of the Natural killer cells towards the pores of the semi permeable membrane. The cells were fixed on the semi permeable membrane of the cell inserts by 4% paraformaldehyde and stained with DAPI (blue).

**Supplementary Table 1.** Cell lines and medium used for culturing

| **Cell line** | **Medium (Additives)** | **Catalogue/Reference Number** | **Manufacturer** |
| --- | --- | --- | --- |
| UM-HACC-2A (Cat T8326)  Optimized salivary gland medium is composed of following ingredients **→** | PriGrow III | TM003 | abm |
|  | Fetal Bovine Serum | A3161001 | Thermo Fisher Scientific |
|  | L-glutamine | Cat 103579-100 | Agilent |
|  | Hydrocortisone 21-hemisuccinate | Cat 74142 | Stem Cell Technologies |
|  | Recombinant human epidermal growth factor | Cat 967812 | R&D systems |
|  | Human recombinant insulin | Cat 91077C-100MG | Sigma Aldrich |
| A-253 (Cat HTB-41) (ATCC) | 1. McCoy’s 5A 2. Fetal Bovine Serum | 1. Cat SLCM0376 2. Cat A3161001 | 1. Sigma Aldrich 2. Thermo Fisher Scientific |
| SCC4 (Cat CRL-1624) (ATCC), SCC9 (Cat CRL-1629) (ATCC), and CAL 27 (CRL-2095) (ATCC). | 1. Dulbecco’s Modified Eagle Medium 2. Fetal Bovine Serum | 1. Cat 11965-092 2. Cat A3161001 | 1. Gibco 2. Thermo Fisher Scientific |
| SCC25 (Cat CRL-1628) (ATCC) and OKF6 (CVCL_L225) | 1. Keratinocyte serum free medium + L-glutamine  2. EGF Human Recombinant  3. Bovine Pituitary Extract | 1. Cat 10724-011 2. Cat 10450-013 3. Cat 13028-014 | 1. Thermo Fisher Scientific 2. Thermo Fisher Scientific 3. Thermo Fisher Scientific |
| Jurkat, Clone E6-1 (TIB-152) (ATCC) | 1. RPMI-1640 2. Fetal Bovine Serum | 1. Cat 11875-093 2. Cat A3161001 | 1. Gibco 2. Thermo Fisher Scientific |
| Natural killer cell, NK-92 (CRL-2407) (ATCC) | 1. Myelocult^TM^ H5100 2. Horse serum heat inactivated 3. Recombinant human Interleukin-2 | 1. Cat 05150 2. Cat H1270 3. Cat 10453-IL | 1. Stem cell technologies 2. Sigma-Aldrich 3. R&D systems |
